# Artificial intelligence in the study of oral lichen planus characteristics: a review

**DOI:** 10.1038/s41405-025-00388-8

**Published:** 2025-12-04

**Authors:** Huishun Yang, Ge Li, Changbin Zhao

**Affiliations:** 1 Department of Periodontics, Shenyang Stomatological Hospital, Shenyang, China; 2 Shenyang Stomatological Hospital Tiexi Outpatient Clinic, Shenyang, China

**Keywords:** Mucositis, Oral medicine

## Abstract

Oral Lichen Planus (OLP) is a common chronic inflammatory disorder with a complex etiology and potential for malignant transformation, posing substantial challenges for clinical diagnosis and management. Conventional diagnostic approaches often depend on clinical experience and subjective assessment, which limits their accuracy and efficiency. Recent advances in artificial intelligence (AI) have introduced novel paradigms for investigating OLP characteristics. This review examines current applications, progress, challenges, and future directions of AI in OLP diagnosis and classification, prediction of disease progression and prognosis, and analysis of molecular features. Through a critical evaluation of existing research, AI demonstrates considerable potential to improve diagnostic objectivity, accelerate clinical decision-making, and reveal underlying disease mechanisms, thereby supporting the development of precision medicine for OLP.

## Introduction

Oral Lichen Planus (OLP) is a chronic inflammatory condition of the oral mucosa, affecting 0.5% to 2% of the global population, with a higher prevalence among middle-aged women [1]. Classified by the World Health Organization (WHO) as an oral potentially malignant disorder (OPMD), OLP presents a risk of progression to oral squamous cell carcinoma (OSCC), though reported malignant transformation rates remain contentious and typically range from 0.2% to 3.5% [2]. The etiology of OLP remains incompletely elucidated but is thought to involve T-cell-mediated autoimmune responses, genetic predisposition, emotional stress, certain dental materials, and medications [3].

OLP exhibits diverse clinical manifestations, including white striations, plaques, papules, erythema, blisters, and erosions. The reticular form is most common, while erosive and atrophic types are frequently painful and substantially impair patients’ quality of life [1]. Due to its variable clinical presentation, OLP is often mistaken for other oral mucosal diseases such as leukoplakia, erythroplakia, and lupus erythematosus, complicating diagnosis and increasing the likelihood of misdiagnosis [4]. Current diagnostic protocols rely mainly on clinical examination and histopathological evaluation, supplemented occasionally by direct immunofluorescence (DIF) to exclude other disorders [5]. Nevertheless, these methods depend heavily on clinician experience and subjective interpretation, resulting in limited accuracy and efficiency [2].

Advances in big data, computational power, and algorithms have enabled artificial intelligence—particularly machine learning (ML) and deep learning (DL)—to demonstrate transformative potential in medical image analysis, disease diagnosis, prognosis prediction, and biomarker discovery [6]. AI systems can autonomously learn and extract discriminative features from large medical datasets, establishing end-to-end mappings from raw data to diagnostic outcomes that enhance objectivity and precision [7]. This review examines the specific applications of artificial intelligence in oral lichen planus research, critically assessing its contributions to diagnostic accuracy, risk assessment, and the elucidation of molecular mechanisms. It further evaluates methodological quality, readiness for clinical translation, and existing implementation barriers.

## Comprehensive and systematic literature search strategy

To ensure the sensitivity and comprehensiveness of the systematic review, the search strategy must combine controlled vocabulary and free-text keywords, cover multiple databases, and achieve precise matching for OLP, AI technologies, and their clinical application objectives.

### Defining research questions and scope based on evidence-based medicine

The scope of literature inclusion will be defined using the standardized PICO framework: P (Population): Patients diagnosed with oral lichen planus (OLP) or oral lichen planus-like lesions (OLLs). I (Intervention/Index Test): Artificial intelligence (AI) and machine learning (ML) methods, including deep learning (DL), natural language processing (NLP), convolutional neural networks (CNN), support vector machines (SVM), Random Forest (RF), and other statistical learning methods. C (Comparator): Traditional diagnostic or predictive methods, such as clinical examination, histopathological assessment, direct immunofluorescence (DIF), or traditional statistical models like Cox proportional-hazards (Cox-PH) nomograms, as well as expert human judgment. O (Outcome): Model performance metrics, including diagnostic accuracy (e.g., sensitivity, specificity, area under the curve [AUC]), lesion classification performance, prediction of malignant transformation risk, prognostic prediction of disease recurrence or progression, and discovery of molecular biomarkers.

### Development of search strategies for multiple databases

The search strategy will employ a modular structure, utilizing Boolean operators to connect different search terms, and will be implemented across several major biomedical and engineering databases, including but not limited to PubMed/MEDLINE, Embase, Web of Science, and Scopus. The search syntax must be highly sensitive to ensure coverage of all relevant AI/ML technical terms, combined with OLP-specific disease nomenclature, to achieve high recall. Given that AI research in OLP involves multimodal data, including clinical images, histopathology, genomics, proteomics, and microbiome data, the search strategy must be expanded by incorporating the following keywords to ensure comprehensive coverage of relevant studies: Data types: “Image Recognition” OR “Histopathology” OR “Digital Slide” OR “Genomics” OR “Proteomics” OR “Microbiome” OR “Multi-omics”; Specific algorithms: SANN OR Xception OR MobileNetV2 OR Mask R-CNN OR Transformer (specific AI models mentioned).

### Screening, inclusion criteria, and data extraction plan

The inclusion criteria will focus on original studies that developed, internally validated, or externally validated AI/ML prediction models (excluding purely conceptual reviews or simple statistical analyses). Emphasis will be placed on models targeting OLP or OLP-related outcomes, such as malignant transformation or recurrence. Exclusion criteria include animal studies, ex vivo experiments, unpublished conference abstracts, and literature unrelated to the development or validation of AI models Table 1. Data extraction will capture technical parameters critical for bias assessment. These parameters include: algorithm type (e.g., CNN, SANN, LLM), size and source of the training dataset, performance metrics (e.g., AUC, sensitivity, specificity, calibration statistics), type of validation (apparent, internal, or external), and detailed information on demographic and clinical characteristics used for model training, which is essential for evaluating generalizability and fairness.

**Table 1:** Performance and Methodology Comparison of OLP Diagnostic Key AI Research.

Author/Year	Dataset Size (OLP/Comparison)	Data type	AI model	Performance Indicators (AUC/Accuracy/F1)	Advantages/Limitations/Validation Status
Achararit et al. 2023 [11]	609 / 480	Clinical image	Xception/MobileNetV2	Acc 82–88%; F1 (Xception Best)	Advantage: Compared multiple CNN models. Limitations: Internal validation only; Lack of critical discussion on sample size, data balance, or external generalization ability.
Ramesh et al. 2025 [17]	202 (OL)/457 (other white lesions)	Clinical image	Xception/MobileNetV2	Acc 89–92%; F1 0.89–0.92 (MobileNetV2 best)	Advantage: Proficient in distinguishing between OL and other white lesions, including OLP. Limitations: Internal validation only; The performance degradation of external validation has not been evaluated [16].
Yu et al. 2024 [2]	N/A (standard test)	Text/Image	ChatGPT-4O, Claude Opus	Recognition rate of 50% -80% (after training)	Advantage: Evaluate the application of LLM in oral pathology. Limitations: Poor performance in rare areas; Belonging to a general model, its performance is significantly inferior to specific AI visual models [1].
Nosratzehi et al. 2018	*N* = 130 (retrospective cohort)	Histopathology (H&E)	ANN	AUC 0.982–0.988；Acc 94.62%	Advantage: It achieves objective quantification of diagnosis and reduces inter observer differences in pathology. Limitations: The sample size is relatively small; Retrospective study; Lack of evaluation of the quality of methodology and external validation.
Toader et al. 2025 [24]	80	Clinical data + DIF	ANN	Test set Acc 85%; Verification set Acc 71%	Limitations: The performance of the test and validation sets has significantly decreased, indicating overfitting or generalization failure of the model [15]. Its clinical applicability needs to be carefully evaluated.

### Control of bias risk and reporting standards

A rigorous evaluation of AI efficacy must be founded upon methodological robustness. To promote transparency and reproducibility in medical imaging AI research, the updated 2024 Checklist for Artificial Intelligence in Medical Imaging (CLAIM) should be employed [2]. CLAIM comprises 44 items designed to guide authors in comprehensively reporting critical information, such as the explicit level of data partitioning (patient-level or image-level), study design (prospective or retrospective), and the availability of models and data [2]. Existing OLP studies are often hampered by incomplete reporting, which impedes effective methodological critique. For algorithms involving the prediction of OLP malignant transformation risk or recurrence, the Prediction model Risk Of Bias Assessment Tool should be utilized to assess their quality and applicability, thereby ensuring clinical reliability [4]. Performance metrics, such as accuracy, F1-score, and the area under the curve (AUC), must be discussed within context. For instance, in external validation datasets, the model’s area under the receiver operating characteristic curve (AUROC) decreases by an average of -0.037, with nearly half of the studies (49.5%) experiencing an AUROC reduction exceeding 0.05 [5]. This indicates that unvalidated high-performance metrics should be considered preliminary findings, and the model’s generalizability must be cautiously evaluated [6]. Methodologies should rigorously assess the strategies employed for managing class imbalance (e.g., common versus rare OLP subtypes) and emphasize the importance of the F1-score and AUC over sole reliance on accuracy [8].

## AI in the diagnosis and classification of oral lichen planus

Early diagnosis of oral lichen planus (OLP) remains challenging, traditionally relying on clinical experience and subjective assessment. Artificial intelligence (AI) now provides new pathways for objective and rapid diagnosis, especially through image recognition and histopathological analysis [2].

### Clinical and histopathological features of OLP

OLP is a chronic inflammatory disorder that occurs predominantly in middle-aged women [1]. Its clinical manifestations are diverse and may involve the oral mucosa, skin, nails, scalp, and other sites [9]. Oral lesions typically present on the buccal mucosa, dorsal and ventral tongue, gingiva, and lips, often with bilateral symmetry [1]. Characteristic histopathological findings include saw-toothed rete ridges, liquefactive degeneration of the basal layer, a dense band-like lymphocytic infiltrate (mainly T-cells) in the subepithelial layer, Civatte bodies, and the absence of epithelial dysplasia or signs of malignancy [1]. Direct immunofluorescence (DIF) aids in differential diagnosis, particularly when distinguishing OLP from pemphigus or pemphigoid. However, intense inflammation in OLP lesions can degrade immune deposits and lead to false-negative DIF results [10].

### Image-based diagnostic models

AI has advanced image-based diagnosis of OLP considerably, largely through deep learning applied to clinical and histopathological images. Early diagnosis has been difficult due to reliance on subjective clinical evaluation [2]. AI techniques, particularly convolutional neural networks (CNNs), are now widely employed for objective and efficient image-based diagnosis [11]. Studies indicate that AI platforms markedly enhance diagnostic accuracy after training on OLP-specific features [2]. For instance, one comparative study assessed the diagnostic performance of ChatGPT-4O, ChatGPT (Diagram-Date extension), and Claude Opus in recognizing OLP². Following OLP-focused training, recognition rates improved substantially: ChatGPT-4O increased from 59% to 77%, ChatGPT (Diagram-Date) from 68% to 80%, and Claude Opus from 15% to 50% [2]. Pretrained recognition rates were higher in common sites such as the buccal mucosa, reaching 94%, 93%, and 56%, respectively [2]. Performance was weaker, however, in rare locations like the gingiva and in complex cases, underscoring limitations in generalizing to atypical presentations. Another investigation applied a deep CNN to clinical images from 609 biopsy-confirmed OLP cases and 480 non-OLP cases [12]. Results demonstrated that all selected CNN models could diagnose OLP and non-OLP lesions, with the Xception model achieving the highest overall accuracy (82–88%) and F1-score [12]. The MobileNetV2 model attained 92% accuracy and F1-scores between 0.91 and 0.92 in discriminating oral leukoplakia and other white lesions (including OLP), outperforming Xception, which reached 89% accuracy and an F1-score of 0.89 [8]. Semantic segmentation models such as SegFormer are also under development for automated lesion segmentation in oral mucosal diseases, offering objective and quantitative diagnostic support. Combined architectures like Mask R-CNN with Swin Transformer have shown promising outcomes in OLP detection, achieving an F1-score of 0.825 and an AUC of 0.948 [13].

OLP exhibits clinical and histopathological overlap with other lichenoid lesions, contributing to considerable inter-observer variability among pathologists [14]. This variability complicates both mechanistic understanding and evaluation of malignant potential. AI has been harnessed to develop machine learning-based artificial neural networks (ANNs) that identify and quantify mononuclear and granulocytic cells in H&E-stained digital slides [14]. This approach reliably detected OLP cases using inflammatory and mononuclear cell counts, yielding AUCs of 0.982 and 0.988, respectively [15]. With a mononuclear cell count cutoff, sensitivity reached 100% and accuracy 94.62% [16]. The method reduces subjectivity, is straightforward to implement in clinical and laboratory environments, and demonstrates good reproducibility [15], indicating that AI could function as a reliable standalone tool for OLP diagnosis.

### Natural language processing in clinical documentation

Natural Language Processing (NLP), a subfield of AI, enables machines to interpret and process human language. Its use in healthcare—particularly for analyzing unstructured data in Electronic Health Records (EHRs)—is expanding [17]. An estimated 80% of medical documentation comprises unstructured data, which has historically been challenging to extract and analyze computationally [18]. NLP engines can extract meaningful information from such data, identifying patient conditions that were previously overlooked or miscoded, which is vital for disease discovery and risk adjustment [16]. In OLP, NLP can analyze clinical notes describing symptoms, medical history, medications, and patient-reported outcomes, thereby supporting comprehensive diagnosis. For example, by quantifying subjective symptoms like pain, roughness, or changes in taste, NLP can help correlate these features with clinical and pathological findings [17]. Furthermore, NLP automates time-consuming documentation tasks, alleviating EHR-related burden on clinicians and freeing them to focus more on patient care. As the volume of clinical documents increases, NLP accuracy will continue to improve, facilitating more customized and precise medical decision-making.

### AI in predicting progression and prognosis of OLP

The chronic nature and potential for malignant transformation of OLP make prognostic prediction a key clinical challenge [19]. Artificial intelligence provides unique advantages in analyzing multidimensional data and identifying complex patterns, offering powerful tools for predicting disease progression, malignant transformation risk, and treatment response in OLP.

### Malignant transformation risk prediction

OLP is recognized by the WHO as an oral potentially malignant disorder (OPMD), with malignant transformation representing a major clinical concern [19]. Although reported transformation rates vary, several risk factors have been established, including smoking (OR = 4.62), alcohol consumption (OR = 3.22), hepatitis C infection (OR = 3.77), and atrophic or erosive OLP subtypes (OR = 2.70) [20]. Conventional risk prediction methods such as Cox proportional hazards (Cox-PH) nomograms, have demonstrated moderate accuracy in predicting malignant transformation in OPMDs, including OLP [21]. However, machine learning algorithms, particularly the Self-Attention Artificial Neural Network (SANN) model, have surpassed traditional statistical approaches [22]. In one study, the SANN model achieved an AUC of 0.9877, with sensitivity, specificity, accuracy, and precision all exceeding 0.96, outperforming Cox-PH nomograms (AUC 0.880–0.902) [23].

Machine learning risk models for oral leukoplakia and oral lichenoid lesions, including OLP, have also yielded promising results, with optimized models using 26 and 15 predictors attaining 97% and 94% accuracy, respectively [23]. External validation demonstrated 100% sensitivity, 88% specificity, and an F1-score of 0.67 in identifying patients who developed malignancy [12]. These findings suggest that AI can effectively integrate demographic, clinicopathological, and treatment data to support risk stratification across diverse clinical contexts.

### Disease recurrence risk assessment

OLP is a chronic condition characterized by recurrent flares that necessitate long-term follow-up [24]. Although direct applications of AI in predicting OLP recurrence remain limited, insights from fields such as oncology indicate considerable potential. Deep learning has been employed to predict postoperative recurrence risk in pediatric low-grade gliomas (pLGGs) by integrating clinical features, MRI-derived deep learning features, and multimodal data [25]. A multimodal model achieved a concordance index (C-index) of 0.85, surpassing models based solely on clinical (0.72) or deep learning MRI (0.79) features [25]. Similarly, deep learning-based multi-omics integration frameworks have predicted breast cancer recurrence risk using gene expression, copy number variation, mutation, and microRNA data [26]. These advances imply that integrating clinical, imaging, and molecular data from OLP patients could enable accurate recurrence prediction tools, thereby facilitating personalized follow-up and intervention strategies.

### Treatment response prediction

There is no cure for OLP, and management focuses on symptom control and preventing progression⁷. Predicting individual therapeutic response is essential for personalized care. Machine learning is increasingly being explored to predict treatment outcomes and tailor therapeutic strategies [27]. For instance, an artificial neural network (ANN) model integrating direct immunofluorescence (DIF) results and clinical data achieved 85% accuracy in test samples and 71% in validation samples for OLP diagnosis, identifying stress, sublingual leukoplakia, and mandibular gingival erosions as important predictors [28]. In broader medical applications, deep learning models have predicted cancer drug response by integrating tumor and drug information without relying on predefined biomarkers [29]. Likewise, AI-driven proteomic studies have identified plasma protein signatures for disease diagnosis and stratification [30]. Applying similar approaches to OLP could enable prediction of responses to topical corticosteroids, photodynamic therapy, or novel agents such as JAK inhibitors based on genomic, proteomic, microbiome, and clinical data [7], thus optimizing treatment selection and improving patient compliance.

### AI in molecular characterization of OLP

The etiology and pathogenesis of OLP remain incompletely elucidated but are known to involve complex immune responses and molecular alterations [3]. Artificial intelligence, integrated with high-throughput omics technologies, is advancing the characterization of molecular features in OLP, thereby supporting biomarker discovery and precision medicine [31].

### Genomic and transcriptomic analysis

Genomic and transcriptomic studies seek to identify genetic variations and expression profiles associated with the disease [32, 33]. Next-generation sequencing (NGS) has enabled culture-independent analysis of the oral microbiome while reducing costs [34]. In OLP, NGS has facilitated comparisons of host gene expression and microbial composition between patients and healthy controls [35]. These investigations have revealed distinct host transcriptional signatures and altered microbial profiles in OLP, including activation of the hepatocyte nuclear factor alpha (*HNF4A*) network and associations with potential pathogens such as *Fusobacterium nucleatum*, *Prevotella melaninogenica*, *Streptococcus intermedius*, and *Streptococcus oralis* [18]. MicroRNAs (miRNAs) contribute to post-transcriptional regulation and have been implicated in OLP pathogenesis [36]. One study reported 24 differentially expressed miRNAs and 2694 regulated transcripts in OLP mucosal tissues, uncovering miRNA–mRNA pairs linked to inflammatory and precancerous processes [37]. Deep learning methods offer improved prediction of genotype–phenotype relationships by capturing non-linear effects and higher-order interactions in gene expression data [22, 38].

### Proteomic studies

Proteomics encompasses the large-scale analysis of proteins, including their isoforms, polymorphisms, and post-translational modifications [39]. Two-dimensional electrophoresis combined with mass spectrometry has identified differentially expressed proteins in OLP tissues, saliva, and serum relative to healthy samples [39, 40]. Although these studies often rely on small sample sizes, they provide a foundation for future biomarker research [41]. Machine learning approaches are increasingly applied in proteomics, such as in predicting protein subcellular localization [42]. Platforms like ProteomicsML streamline the acquisition of proteomic data for machine learning, enhancing reproducibility and accessibility [43]. AI-guided proteomic analyses have successfully diagnosed and stratified diseases based on plasma protein signatures [43, 44], indicating similar promise in OLP.

### Oral microbiome research

The oral microbiome is increasingly recognized as a factor in OLP pathogenesis [3]. Studies indicate dysbiosis in the oral microbial communities of OLP patients, with more marked alterations observed in the buccal mucosa than in saliva [3]. Although consistent taxonomic shifts have not been identified across studies, functional changes in the microbiome may hold greater relevance than compositional differences alone [13]. NGS-based analyses have associated OLP with several potential pathogens, including *F. nucleatum*, *P. melaninogenica*, *S. intermedius*, *S. oralis*, and *Prevotella denticola* [18]. Some of these microbes may activate host immune pathways such as those mediated by *HNF4A* [18]. AI and machine learning can decipher complex microbiome data, uncover correlations with clinical features or treatment response, and integrate multi-omics data to construct holistic models of OLP pathogenesis [45].

### Biomarker discovery

Biomarkers are essential for early detection, diagnosis, prognosis, and treatment selection [46]. AI is particularly adept at recognizing patterns in high-dimensional data that evade conventional statistical methods. Multi-omics integration combines genomic, transcriptomic, proteomic, metabolomic, lipidomic, and epigenomic data with clinical variables to furnish a comprehensive perspective on disease mechanisms [47]. AI models can detect molecular patterns indicative of disease onset or progression. For instance, integrating genomic variants with proteomic and metabolomic profiles has enabled early detection of cancer and other complex diseases [47, 48]^.^ In neurodegenerative disorders, machine learning has identified diagnostic biomarker panels involving liver-derived molecules, corroborating the liver–brain axis hypothesis [49]. Although AI-assisted multi-omics biomarker discovery in OLP is still nascent, successes in other disciplines suggest considerable potential. AI may help identify molecular signatures correlating with disease activity, malignant transformation risk, or therapeutic response in OLP, paving the way for tailored treatments with improved efficacy and reduced adverse effects [49, 50].

### Challenges, future directions, and outlook

Despite its promise, the widespread application and clinical translation of AI in OLP research face several challenges.

### Data quality, standardization, and class imbalance

The performance of artificial intelligence (AI) models is highly dependent on high-quality, large-scale, and diverse training data. In healthcare, data acquisition is costly, privacy protection is stringent, and standardization is often insufficient, leading to limited datasets for oral lichen planus (OLP).

#### Standardization of clinical records

The clinical adaptability of AI in dental medicine strongly depends on improving patient data collection and standardizing clinical records [24, 41]. Current technical obstacles include inconsistent terminology and isolated Electronic Health Record (EHR) systems. Low-quality data, such as duplicate records or inaccurate/incomplete details, may lead AI models to erroneous conclusions. Therefore, structured and standardized numerical data input must be established at the provider level to ensure data consistency. For instance, AI platforms often underperform in cases involving rare sites and complex presentations, which reflects insufficient diversity in the training data. Data augmentation techniques, including deep generative models such as Variational Autoencoders (VAEs), Generative Adversarial Networks (GANs), and diffusion models, may help generate more realistic and diverse training data. However, their ability to simulate real-world complexity requires further validation [51]. Model generalization remains a core challenge—models trained on specific datasets often perform poorly on data from different populations, institutions, or device sources [52]. Establishing large-scale, multi-center, multi-ethnic standardized datasets and developing more robust models are necessary to ensure their reliability in real-world clinical settings.

#### Class imbalance problem

Datasets in existing studies often exhibit class imbalance (e.g., common OLP subtypes versus rare malignant transformation cases), which may cause models to be biased towards the majority class and reduce their sensitivity to high-risk minority classes. Strategies to address this include:

Data-level approaches: Increasing the sample size of minority classes through techniques such as the Synthetic Minority Over-sampling Technique (SMOTE) or image augmentation. 2. Algorithm-level approaches: Applying cost-sensitive learning or specialized loss functions (e.g., Focal Loss) to assign higher weights to minority classes during the training process.

### Interpretability challenges and clinical applicability

#### Explainable artificial intelligence (XAI)

The characteristics of oral lichen planus (OLP) are multidimensional, involving clinical, histopathological, molecular (genomics, proteomics, microbiome), and systemic factors such as smoking, alcohol consumption, and HCV infection [9]. Single-modality data analysis can only provide partial insights [53]. Multimodal data integration represents a crucial future direction; constructing more comprehensive disease models can enhance diagnostic accuracy, prognostic prediction, and treatment response assessment capabilities [23]. However, integrating heterogeneous multimodal data presents technical challenges, necessitating the development of advanced fusion algorithms. Furthermore, AI models, particularly deep learning, are often regarded as “black boxes” with limited transparency and interpretability [50, 54]. In clinical practice, physicians need to understand the AI decision-making process to trust and adopt its recommendations [55]. The development of explainable artificial intelligence (XAI) to untangle key features and decision logic is essential for the clinical adoption of AI for OLP [56]. Specific XAI techniques, such as gradient visualization methods (e.g., Grad-CAM, NDG-CAM) and perturbation analysis methods (e.g., LIME, SHAP), can highlight precise regions within histopathological images that influence model decisions [2, 7, 8, 13], thereby enhancing model transparency and auditability.

#### Clinical translation and benchmarking against human experts

The evaluation of AI systems’ clinical translational capabilities remains insufficient, and comparisons with human expert performance are limited. Direct comparisons between AI performance and human expert diagnostic capabilities are required. Studies indicate that while certain Convolutional Neural Networks (CNNs) achieve performance comparable or superior to human experts in controlled environments, general-purpose large language models (LLMs) demonstrate significantly inferior performance in diagnosing rare, domain-specific oral pathologies (e.g., achieving an accuracy of only 40.0% for certain oral lesions compared to 83.3% for human experts). AI in dentistry remains primarily in the research phase and has not been fully integrated into routine practice. Challenges include distrust in “black box” reasoning, fear of loss of autonomy, concerns regarding infrastructure requirements and implementation costs, as well as insufficient acceptance and trust among clinicians towards AI recommendations.

### Ethical, legal, and regulatory barriers

The application of AI in medicine raises significant ethical and regulatory concerns in following areas:

1. Data Privacy and Security: Given the processing of highly sensitive patient health information, including clinical images, omics data, and EHR text, data privacy and security are paramount concerns. Ensuring data anonymization, de-identification, and secure storage is essential to maintain patient trust and comply with regulatory standards. It is particularly noteworthy that hybrid and multimodal AI systems, which integrate data from diverse sources, significantly amplify the complexity of ethical and legal challenges. This necessitates that informed consent procedures be adapted to address this complexity.
2. Algorithmic Bias and Fairness: Algorithmic bias refers to the phenomenon where an AI model exhibits disproportionately lower performance for specific demographic groups (e.g., racial or gender minorities) due to systematic errors in the training data. This issue is highly relevant to the performance limitations of AI models in detecting rare lesions or lesions in atypical locations. Such bias often originates from the historical underrepresentation or complete absence of certain groups within biomedical datasets, causing AI models to potentially reinforce existing societal prejudices. This, in turn, may lead to misdiagnosis or exacerbate healthcare disparities. Primary strategies to ensure fairness involve rigorous external validation on diverse, multi-center, and multi-ethnic datasets, coupled with the promotion of open science practices and inclusive data standards.
3. Legal Liability and Accountability: The use of AI-assisted decision-making in medicine raises critical legal questions: in cases of erroneous AI-assisted diagnosis or prognosis, who should be held accountable—the developer, the healthcare institution, or the clinician? The determination of liability and compensation is complicated by factors such as the varied applications and intended users of AI systems, as well as their inherent “black box” nature. Current legal standards mandate that clinicians cannot blindly rely on AI tool outputs; they must exercise appropriate oversight and independent professional judgment. Consequently, clinicians remain liable for diagnostic errors that cause patient harm. The current lack of a clear legal framework defining the responsibilities of developers and hospitals hinders the broader adoption of AI by clinicians.
4. Regulation and Continuous Learning: AI tools in oral medicine are typically classified as Software as a Medical Device (SaMD). The FDA (Food and Drug Administration) encourages the development of safe and effective AI medical devices, requiring them to meet appropriate premarket pathways (e.g., 510(k) or PMA). The FDA’s traditional medical device regulatory model was not designed for adaptive AI/ML technologies, as these algorithms possess the capability to continuously learn and improve their performance from real-world use and experience. This continuous learning can lead to performance changes post-deployment, thereby posing regulatory challenges. Consequently, the FDA is exploring a tailored regulatory framework for AI/ML-based SaMD that emphasizes total product lifecycle management and continuous monitoring to ensure ongoing safety and effectiveness. Furthermore, regulatory bodies in regions such as Europe are also establishing post-market surveillance (PMS) requirements for “continuously learning AI systems” to ensure the safe use of high-risk AI systems.

## Conclusions and future perspectives

Artificial intelligence (AI) demonstrates significant potential in advancing both the research and clinical management of oral lichen planus (OLP), particularly in image-based objective diagnosis and the prediction of malignant transformation risk. However, the lack of methodological rigor, external validation, and contextual discussion of performance comparisons in existing studies represents major obstacles hindering its clinical translation. Future efforts should focus on the following areas:

1. Development of Large-Scale, Standardized Databases: Overcoming the challenges of data scarcity and insufficient diversity is crucial. Efforts should be dedicated to improving the standardization of patient data collection and clinical documentation to support the generalizability and clinical applicability of AI models.
2. Mandatory External Validation and Reporting Standards: External validation must be mandated as a compulsory requirement to ensure the reliability of AI models in real-world settings. Strict adherence to the CLAIM 2024 updated guidelines and the criteria is essential to guarantee study transparency and reproducibility.
3. Integration of Explainability: Commitment to the development and integration of Explainable AI (XAI) techniques (e.g., Grad-CAM, LIME) is necessary to enhance model transparency, foster clinical trust, and ensure safety and auditability.
4. Clarification of Regulatory and Liability Frameworks: Establishing robust ethical, legal, and regulatory (ELR) frameworks is imperative to address issues related to data privacy, algorithmic bias, and legal liability, thereby ensuring the safety, efficacy, and equity of AI-based medical devices.

As AI transitions from research to clinical integration, sustained collaboration among healthcare professionals, data scientists, and regulatory bodies is essential. By addressing these challenges, AI is poised to become an indispensable tool in oral medicine, enabling early and accurate diagnosis, precise prognostic prediction, and personalized treatment for patients with OLP.
